# Are There Differences in Motor Coordination Among Spanish Primary School Students?

**DOI:** 10.3390/jfmk10030275

**Published:** 2025-07-17

**Authors:** Ricardo Fernández-Vázquez, Martín Barcala-Furelos, Javier Cachón-Zagalaz, Víctor Arufe-Giráldez, Marcos Mecías-Calvo, Rubén Navarro-Patón

**Affiliations:** 1 Faculty of Humanities and Educational Sciences, University of Jaén, 23071 Jaén, Spain; ricardofervaz28@gmail.com (R.F.-V.); jcachon@ujaen.es (J.C.-Z.); 2 Faculty of Education Sciences, Universidade de Santiago de Compostela, 15782 Santiago de Compostela, Spain; martin.barcala@usc.es; 3 Research Group on Motor Skills, Physical Education, and Health, Universidade de Santiago de Compostela, 27001 Lugo, Spain; marcos.mecias@usc.es (M.M.-C.); ruben.navarro.paton@usc.es (R.N.-P.); 4 Research Group in Technology Applied to Occupational, Equality and Health Research, Faculty of Education, University of A Coruña, 15008 A Coruña, Spain; 5 Facultade de Formación do Profesorado (Campus Terra), Universidade de Santiago de Compostela, 27001 Lugo, Spain

**Keywords:** schoolchildren, locomotor control, visuomotor control, motor development

## Abstract

**Background:** Motor coordination is a fundamental skill in childhood. Factors such as age, sex, and regular sports practice influence its development. However, there is little research that jointly analyzes the impact of these factors on the motor skills and abilities of primary school children. The objective of this study was to analyze what happens to different motor skills and abilities (i.e., locomotor coordination (LC); visuomotor coordination (VC); foot object control coordination (FOCC); hand object control coordination (HOCC); global motor coordination (GMC)) in relation to regular and regulated sports practice (yes vs. no), sex (boys vs. girls), and age (6 to 11 years) in a sample of 663 primary schoolchildren (8.59 ± 1.65 years; 48.26% boys) from Galicia (Spain). **Methods:** The 3JS test was used to analyze motor coordination. To determine differences between the 3JS variables, a multivariate analysis of covariance (MANCOVA) was performed based on age, sex, and sports practice, including a BMI category (normal weight, overweight, or obese) as a covariate to avoid potential confounding factors. **Results:** Statistically significant differences were observed based on age [LC (*p* < 0.001); VC (*p* < 0.001); FOCC (*p* < 0.001); HOCC (*p* < 0.001); CMG (*p* < 0.001)], sex [i.e., VC (*p* < 0.001); FOCC (*p* < 0.001); HOCC (*p* < 0.001); CMG (*p* < 0.001)], and sports practice [i.e., LC (*p* < 0.001); VC (*p* = 0.008); HOCC (*p* < 0.001); CMG (*p* < 0.001)], after the application of the 3JS battery. **Conclusions:** Locomotor coordination in Primary Education is modulated by the interaction between age, sex, and sports practice. All of these variables increase with age, with higher scores in boys than in girls, and higher scores in children who participate in sports than in those who do not.

## 1. Introduction

Motor coordination (MC) is an essential capacity for full human development, both physical and cognitive [1]. It depends on the neurological structure and is responsible for controlling skeletal muscles by mobilizing and directing the muscles necessary to perform a motor action in an orderly, precise, and efficient manner [2]. MC is essential in Primary Education as it helps students develop motor skills that allow them to perform basic motor skills, such as crawling, walking, running, throwing, or receiving [3]. Similarly, it improves motor performance and behavior [4], which, in later stages, will facilitate the learning of motor skills specific to the field of sports [5]. In this sense, motor competence can be understood as the ability to perform different actions, including gross motor coordination (locomotor) and/or fine motor coordination (object control) [6]. The development of motor competence is also necessary to integrate different motor skills, both at the level of locomotor coordination and object control (hand-foot) [7,8,9].

MC evolves throughout life, although its development does not occur uniformly [10], and can be influenced by various factors such as age, sex [11,12], body composition [13], physical activity levels [14], sports practice [15], or other more abstract variables such as motor competence [14], or the relative effect of age [12]. Therefore, it is necessary to understand how these factors can contribute to improving MC, generating healthier development in Primary Education students [16].

Regarding age, scientific evidence indicates that Primary Education (6–12 years) is an optimal period for the proper acquisition of motor skills [2]. As students age, their coordination skills improve, although this increase does not occur equally at each age [9]. These variations may be due to the difference in biological age between the sexes, since girls show a faster maturation rate than boys [17]; therefore, between 8 and 9 years of age, they may present greater motor skills than boys [18]. Furthermore, coordination skills do not evolve homogeneously, since students tend to obtain better results in actions that involve controlling objects with the hand, compared to those performed with the foot, and this difference increases in older students of both sexes [8].

Considering the influence of sex on the development of MC, boys generally present a higher level of gross motor skills (locomotor skills) and girls a better performance in fine motor skills (manipulative skills) [19]. Despite this, there are studies that observe that girls show better locomotor coordination capacity than boys, and that boys demonstrate greater coordination capacity in object control (especially with the foot) than girls [8]. Other studies show that boys obtain better indices of general motor coordination, locomotor coordination, and coordination in object control than girls [20].

Another factor that directly influences the improvement of motor coordination is the level of motor practice [2]. In this sense, boys tend to be more active than girls [17]. Despite this, physical activity levels in the child population, regardless of sex, are insufficient [13], which results in a reduction in their general motor coordination indices and, consequently, a lower level of motor mastery, which ultimately leads to sedentary behaviors [20]. Therefore, the importance of developing coordination skills transcends the school environment, making participation in extracurricular sports activities essential [5]. Regular physical activity contributes to more complete motor and postural development, promoting psychomotor development and improving posture and musculoskeletal health. However, it is essential to ensure that the type of sport and duration of practice take into account the prevention of possible postural disorders [21]. In this regard, the study by Puszczalowska-Lizis et al. [21] on young female volleyball players highlights that while this sport has a beneficial effect on the formation of thoracic kyphosis, it can also lead to a flattening of the lumbar lordosis due to specific training loads. This finding underscores the importance of a holistic approach to sports training, in this specific case, but which can be applied to other sports. Therefore, exercises should be incorporated to strengthen the stabilizing muscles of the lumbar spine, improve spinal mobility, and control anterior pelvic tilt. Another aspect to consider is that, for example, there may be postural differences between those who practice certain sports and those who do not, as is the case in the study by Puszczalowska-Lizis et al. [21], which found that female athletes presented a smaller lumbar curvature compared to their untrained peers, which could affect the cushioning capacity of the spine and increase the risk of musculoskeletal overload. Therefore, these factors must be taken into account when measuring motor aspects such as motor coordination, the subject of this research. This reinforces the need to regularly assess young athletes’ posture and adapt their training routines to ensure balanced and sustainable physical development.

Improving coordination skills and establishing a solid foundation of motor skills will allow students to enjoy physical activity both inside and outside of school, generating active and healthy lifestyle habits [5]. Physical education should implement the necessary tools to identify the factors that directly influence the development of MC, taking into account the individual characteristics of each student [5]. The 3JS test [22] is an interesting tool to determine both the level of global coordination of students, as well as their levels of locomotor coordination and coordination in object control (hand-foot) (i.e., locomotor coordination (LC); visuomotor coordination (VC); foot object control coordination (FOCC); hand object control coordination (HOCC); global motor coordination (GMC)).

To the authors’ knowledge, age, gender, and regular or sport practice have not yet been analyzed as factors influencing motor skills and abilities in primary school children. Therefore, the objective of this research was to analyze what happens to different motor skills and abilities (LC, VC, FOCC, HOCC, and GMC) in relation to regular and regulated sport practice, gender, and age, in a sample of Spanish primary school students. Based on this objective, we posed the following questions: Do primary school students increase their scores on LC, VC, FOCC, HOCC, and GMC with age? Do boys obtain higher scores than girls on LC, VC, FOCC, HOCC, and GMC? Do students who regularly practice sports present higher scores on LC, VC, FOCC, HOCC, and GMC compared to those who do not?

## 2. Materials and Methods

### 2.1. Study Participants

This is an observational, descriptive, cross-sectional, ex post facto study [23] in which seven publicly funded educational centers from the Xunta de Galicia were invited to participate. Access to the sample was performed non-randomly and by convenience, depending on the accessible centers. Based on information on students enrolled in public educational centers in Galicia (91,373) (Galician Institute of Statistics, 2025 [24]), the sample size was estimated at 661 students, with a confidence level of 99% and a margin of error of 5%. The inclusion criteria for participation in this study were: being between 6 and 11 years old, having signed authorization from their parents or legal guardians, and having consent to participate in the study. The exclusion criteria were that participants did not have musculoskeletal diseases or injuries, upper or lower extremity conditions that would prevent them from performing each of the tests, neurological disorders, or orthopedic surgeries that could affect the results.

### 2.2. Study Variables

The dependent variables, corresponding to the coordination skills [locomotor coordination (LC); visuomotor coordination (VC); foot object control coordination (FOCC); hand object control coordination (HOCC); global motor coordination (GMC)] have been described, comparing them according to sex (boy vs. girl), age (6 to 11 years), and practice of sport (yes vs. no), which were considered as independent variables, using BMI (normal weight; overweight; obesity) as a covariate. The variables of sport type, frequency, and intensity of practice were not controlled. Although chronological age was taken into account, the participants’ pubertal stage and the quality of physical education classes at each center were not assessed.

### 2.3. Tools

An ad hoc questionnaire was used to collect sociodemographic data (age, sex, grade, and extracurricular sports practice).

The 3JS test [22] was used for data collection. This 7-component test (Table 1) assesses the development of motor, visuomotor, and general dynamic coordination through a qualitative procedure of observation and objective evaluation of the execution of the skills developed in each task. Scoring ranges from 1 to 4, with 1 being the most rudimentary way of performing the task and 4 being the most expert [7]. This MC test has been validated for the Spanish population aged 6 to 11 years, with high internal consistency, very high temporal stability, and interobserver agreement [22].

Students complete a course in which they perform 7 consecutive tasks without a break between them. In each task, they develop a different motor task that demonstrates a different type of coordination: three tasks, general dynamic coordination, and four tasks, visuomotor coordination (Table 1). The order of the tests is as follows: 1. vertical jump with both feet together over spikes; 2. jumping upwards and performing a turnabout the longitudinal axis; 3. throwing two balls at a goal post from a distance without leaving the square; 4. hitting two balls at a goal post from a distance without leaving the square; 5. running a slalom; 6. dribbling a basketball back and forth, completing a simple slalom, and changing direction around a pivot; 7. dribbling a ball back and forth with the foot, completing a simple slalom, and changing direction around a pivot.

**Figure 1:**
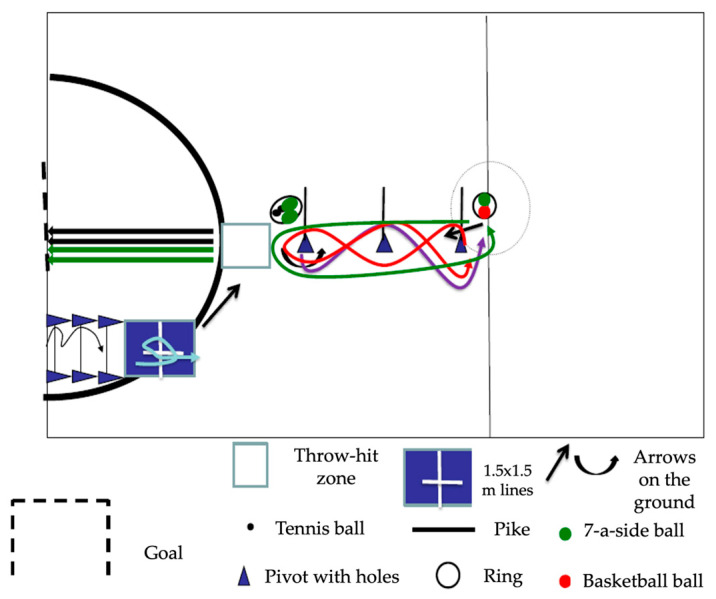
Arrangement of the material for testing the 3JS battery.

The test must be carried out in a 10 × 20 m area on a smooth, even surface (outdoors or indoors; Figure 1). The equipment needed to set up the circuit is 9 cones with holes, 6 cleats, 2 tennis balls, 3 soccer balls, 1 basketball, and 1 mat.

#### Procedure for Applying the Test

Students should be provided with instructions on how to complete the 7 tasks, their order of execution, and the scoring system.

1. Students should be allowed to practice on the circuit before moving on to the final test.
2. Start of the assessment: Once the student has recovered from any fatigue from the previous test, he or she must stand in the starting area (Figure 1) in a static, bipedal position, and the evaluator will stand at the height of the starting box, give the starting signal, and move laterally onto the circuit (Figure 1).
3. Test development: During the test, participants may be reminded of the order of the tasks, but under no circumstances will comments or corrections be made regarding their execution. If a student fails, they must wait two minutes before retaking the test. A student is considered to have failed if they make an error in direction or do not perform any of the tasks in the established order. The evaluator will objectively observe and grade each of the seven course tasks according to the evaluation criteria [7]. The evaluator will record the score for each task.

### 2.4. Ethics

The research was approved by the Ethics Committee of the national platform EDUCA (code 08/2024 on 10 July 2024), under the standards established in the Declaration of Helsinki.

### 2.5. Statistical Analysis

Demographic data were expressed as frequencies for categorical variables (i.e., sex) and as measures of central tendency (mean and standard deviation) for quantitative variables (i.e., age). The internal consistency reliability of the instrument scores was estimated with the McDonald Omega for each motor coordination factor of the 3JS. The Kolmogorov–Smirnov test was used to determine the normal distribution of the data (*p* > 0.05). In cases where the normal distribution was not observed, a robust standard error was used, considering the large sample size of the study, and the bootstrap method was also employed. To determine differences between the 3JS test variables [locomotor coordination (LC); visuomotor coordination (VC); foot object control coordination (FOCC); hand object control coordination (HOCC); global motor coordination (GMC)], sex (boy; girl), age (6–11 years), and regular sports practice (yes/no), a multiple analysis of covariance (MANCOVA) was performed, using the Bonferroni statistic to determine the significance of the interaction between the study variables, while eta squared (η^2^) was used to determine the effect size. BMI (normal weight, overweight, or obese) was used as a covariate to control for potential confounders. Statistical analyses were performed using SPSS software (v. 28, IBM Corporation, New York, NY, USA). Statistical significance was set at *p* < 0.050.

## 3. Results

A total of 663 healthy primary school children from Galicia (Spain) were evaluated. A total of 320 (48.26%) were boys and 343 (51.74%) were girls, with a mean age of 8.59 (SD = 1.65). A total of 499 (75.27%) participated in sports, while 164 (24.73%) did not. The age distribution of participants was 94 (14.18%) for 6-year-olds, 99 (14.93%) for 7-year-olds, 104 (15.68%) for 8-year-olds, 132 (19.80%) for 9-year-olds, 127 (19.15%) for 10-year-olds, and 107 (16.26%) for 11-year-olds. The distribution by BMI was normal weight 396 (59.7%), overweight 160 (24.15%), and obesity 107 (16.15%). Table 2 shows the results overall, by age and sex, and physical activity practice in each of the dimensions of motor coordination that have been evaluated using the 3JS test battery.

### 3.1. Locomotor Coordination Results

The results of the MANCOVA for locomotor coordination (LC, Table 3) indicated a significant main effect of the age factor [F (5, 638) = 29.908, *p* < 0.001, η^2^ = 0.190], which increased progressively as the age of the students increased. A significant main effect was also found for the factor of practicing sport [F (1, 638) = 18.586, *p* < 0.001, η^2^ = 0.028], where children who practiced sport presented higher scores than children who did not. Finally, a statistically significant age × sport interaction was found [F (5, 638) = 2.420, *p* = 0.035, η^2^ = 0.019], where 6-year-old children, whether they practiced sport or not, presented lower scores than the rest of the ages. There are no significant differences in the sex factor (*p* = 0.088), nor in the interaction sex × age (*p* = 0.966), sex × sport (*p* = 0.216), or sex × age × sport (*p* = 0.186).

### 3.2. Visuomotor Coordination Results

The results of the MANCOVA in visuomotor coordination (VC; Table 4) indicated a significant main effect of the sex factor [F (1, 638) = 73.931, *p* < 0.001, η^2^ = 0.104], with higher scores for boys than for girls. There is also a significant main effect for the age factor [F (5, 638) = 47.393, *p* < 0.001, η^2^ = 0.271], which increases progressively as the age of the students increases. A significant main effect was found for the sports practice factor [F (1, 638) = 72.313, *p* < 0.001, η^2^ = 0.102], where children who practiced sports had a higher score compared to children who did not. A statistically significant interaction was also found between sex and age [F (5, 638) = 11.744, *p* = 0.033, η^2^ = 0.019], where boys obtained higher scores than girls at all ages studied; in the interaction of sex × sport [F (1, 638) = 19.652, *p* < 0.001, η^2^ = 0.030], where boys who practiced sports presented higher scores than girls who also practiced them; in the interaction age × sport [F (5, 638) = 2.290, *p* = 0.044, η^2^ = 0.018], where 6-year-old children, whether they practiced sports or not, presented lower scores than the rest of the ages. In addition, there are significant differences at all ages, between children who practice sports and those who do not, with higher scores among those who practice sports (except among 10-year-old boys). Finally, significant differences were found in the interaction of sex × age × sport [F (5, 638) = 2.560, *p* = 0.026, η^2^ = 0.020], where boys who practiced sports presented higher scores at all ages, compared to girls, while the same is not true among children who did not practice sports. Furthermore, boys of all ages who practice sports obtain better scores in this dimension compared to boys who do not. However, the same is not true for girls (except for 6-year-olds).

#### 3.2.1. Foot Object Control Coordination Results

The results of the MANCOVA in the foot object control coordination (FOCC; Table 5) indicated that there was a significant main effect of the sex factor [F (1, 638) = 102.079, *p* < 0.001, η^2^ = 0.138], where boys presented higher scores than girls. There is also a significant main effect for the age factor [F (5, 638) = 26.069, *p* < 0.001, η^2^ = 0.170], which increases progressively as the age of the students increases. A significant main effect was found for the sports practice factor [F (1, 638) = 59.431, *p* < 0.001, η^2^ = 0.085], where children who practiced sports presented higher scores than children who did not practice sports. Finally, a statistically significant interaction was found in the interaction of sex × sport [F (1, 638) = 18.528, *p* < 0.001, η^2^ = 0.028], where boys who practiced sports presented higher scores compared to girls, and in the interaction of sex × age × sport [F (5, 638) = 2.742, *p* = 0.018, η^2^ = 0.021], where boys who practiced sports presented higher scores at all ages compared to girls. No significant differences were observed in the rest of the interactions studied (*p* > 0.050).

#### 3.2.2. Hand Object Control Coordination Results

The results of the MANCOVA in the hand object control coordination (HOCC; Table 6) indicated that there was a significant main effect of the sex factor [F (1, 638) = 22.263, *p* < 0.001, η^2^ = 0.034], where boys obtained higher scores than girls. There is also a significant main effect for the age factor [F (5, 638) = 48.319, *p* < 0.001, η^2^ = 0.275], which increases progressively as the age of the students increases. A significant main effect was found for the sports practice factor [F (1, 638) = 49.776, *p* < 0.001, η^2^ = 0.072], where children who practiced sports obtained higher scores than children who did not. In addition, a statistically significant interaction was found between sex and age [F (5, 638) = 2.623, *p* = 0.023, η^2^ = 0.020], where boys obtained higher scores than girls at all ages studied, except at 11 years old, where the opposite occurred; in the interaction of sex × sport [F (1, 638) = 11.419, *p* = 0.001, η^2^ = 0.018], where boys who practiced sports presented higher scores than girls who also practiced them; in the interaction of age × sport [F (5, 638) = 2.756, *p* = 0.018, η^2^ = 0.021], where children aged 6 and 7 years old presented lower scores than the rest of the ages, whether they practiced sports or not. Furthermore, there are significant differences at all ages, except between children who practice sports and those who do not, at 9 and 10 years old, with higher scores among those who practice sports. No significant differences were observed in the other interactions studied (*p* > 0.050).

**Table 6:** Hand object control coordination results of the 3JS test based on age, gender, and sports practice.

		Boys		Girls		Main Effects			Interactions
	SP	Yes	No	Yes	No	Gender F	Age F	SP F	F
	Age	M ± SD	M ± SD	M ± SD	M ± SD				
HOCC (2–8)	6	4.31 ± 1.27	2.92 ± 1.18	3.96 ± 1.34	2.68 ± 0.88	22.263 ***	48.319 ***	49.776 ***	(2) 11.419 ** (3) 2.756 *
	7	5.50 ± 1.34	3.60 ± 0.54	4.50 ± 1.44	4.07 ± 1.07				
	8	6.05 ± 1.37	5.35 ± 1.00	4.89 ± 1.22	4.82 ± 1.28				
	9	6.71 ± 1.25	5.66 ± 1.27	5.47 ± 1.34	4.88 ± 1.56				
	10	6.73 ± 1.02	6.33 ± 1.00	5.44 ± 1.05	5.35 ± 1.08				
	11	6.82 ± 1.22	4.40 ± 1.14	5.83 ± 1.19	5.55 ± 1.50				

Note. M = Mean; SD = Standard deviation. SP = Sport practice; HOCC = Hand object control coordination. * *p* < 0.05; ** *p* < 0.01; *** *p* < 0.001; Interactions: (1): Gender × Age; (2) Gender × Sports Practice; (3) Age × Sports Practice; (4) Gender × Age × Sport Practice.

### 3.3. Global Motor Coordination Results

The MANCOVA results for global motor coordination (GMC; Table 7) indicated a significant main effect of sex [F (1, 638) = 41.325, *p* < 0.001, η^2^ = 0.061], with boys scoring higher than girls. There was also a significant main effect for age [F (5, 638) = 50.894, *p* < 0.001, η^2^ = 0.285], which increased progressively with the age of the students. Furthermore, a significant main effect was found for the factor sports practice [F (1, 638) = 58.858, *p* < 0.001, η^2^ = 0.084], where children who practiced sports had higher scores than children who did not. Finally, a statistically significant interaction was found between sex and sport [F (1, 638) = 12.206, *p* = 0.001, η^2^ = 0.019], where boys who practiced sports had higher scores than girls who also did, in the interaction of age × sport [F (5, 638) = 2.945, *p* = 0.012, η^2^ = 0.023], where 6 and 7-year-old children who practiced sports and 6-year-olds who did not had lower scores than the rest of the ages. Furthermore, there are significant differences at all ages, except between 10-year-olds who practice sports and those who do not, with higher scores among those who do. No significant differences were observed in the other interactions studied (*p* > 0.050).

**Table 7:** Global motor coordination results of the 3JS test based on age, gender, and sports practice.

		Boys		Girls		Main Effects			Interactions
	SP	Yes	No	Yes	No	Gender F	Age F	SP F	F
	Age	M ± SD	M ± SD	M ± SD	M ± SD				
GMC (2–8)	6	17.40 ± 3.98	13.23 ± 3.13	15.88 ± 3.59	11.42 ± 3.32	41.325 ***	50.894 ***	58.858 ***	(2) 12.206 ** (3) 2.945 *
	7	20.66 ± 3.94	15.60 ± 0.89	17.13 ± 3.82	16.50 ± 3.54				
	8	22.51 ± 3.45	20.21 ± 3.01	18.71 ± 3.11	18.58 ± 3.80				
	9	23.89 ± 3.60	21.23 ± 3.08	19.94 ± 3.68	18.61 ± 3.63				
	10	23.67 ± 2.54	21.55 ± 3.77	20.00 ± 2.95	19.20 ± 3.76				
	11	24.26 ± 3.61	17.00 ± 4.06	20.27 ± 3.28	19.00 ± 3.64				

Note. M = Mean; SD = Standard deviation. SP = Sport practice; GMC = Global motor coordination. * *p* < 0.05; ** *p* < 0.01; *** *p* < 0.001; Interactions: (1): Gender × Age; (2) Gender × Sports Practice; (3) Age × Sports Practice; (4) Gender × Age × Sport Practice.

## 4. Discussion

The objective of this research was to analyze what happens with the different motor skills and abilities (i.e., locomotor coordination (LC); visuomotor coordination (VC); foot object control coordination (FOCC); hand object control coordination (HOCC); global motor coordination (GMC)), in relation to the systematic and regulated sports practice, the sex and age, in a sample of Spanish Primary Education students.

### 4.1. Locomotor Coordination

In the present study, a significant increase in this coordination capacity with age is observed [9,25]. These results may be due to the fact that the development of locomotor coordination is an evolutionary process [26], in which factors such as the maturation of skeletal muscles and the nervous system [2], together with motor experience, favor its correct development during the Primary Education stage. If this does not occur, neurodevelopmental disorders could be associated [27]. In the interaction age × sex, the evolution does not occur in the same way since girls and boys have a different evolutionary calendar of biological age between the infantile and prepubertal stages [17]. Despite this, in the present study, no significant differences were observed in the sex factor, unlike the study by Hurtado Almonacid et al. [20], where, after conducting an evaluation with the 3JS test, it was observed that boys presented better results than girls. This fact raises the need for a more in-depth analysis of coordination domains based on the profile of sports practice, even outside of the educational center [28].

Regarding the interaction between the level of locomotor coordination and participation in sports, this study shows that students who participate in sports obtain better locomotor coordination scores than those who do not [17,29,30]. Along these lines, scientific evidence shows that one of the main factors that contributes to the improvement of locomotor coordination levels is participation in sports activities, both inside and outside of physical education sessions [31]. The optimal practice profiles for the development of locomotor coordination are team [28] or individual sports. On the other hand, 6-year-old students, whether or not they participate in sports, show significantly lower locomotor coordination scores than other ages, as is the case in the study by Cenizo-Benjumea et al. [8], which could mark the reference age in the evolutionary calendar for the development of locomotor coordination [26]. Taking into account the results of this study, it could be affirmed that, in addition to the degree of maturation of the skeletal muscles and the nervous system that occurs from that age onwards [2,32], the need for motor experiences would be essential for its improvement [27,33].

Finally, a significant interaction was found between age and sports practice, with boys obtaining better results in terms of locomotor coordination than girls at all ages analyzed [34], so locomotor coordination increases with age and, to a greater extent, in boys than in girls.

### 4.2. Visuomotor Coordination

In this dimension, a tendency towards a progressive increase in visuomotor coordination scores is observed as the age of the schoolchildren increases, as in the study carried out by Cenizo Benjumea et al. [8], who reflect a progressive improvement in coordination throughout age, showing better results in object control coordination compared to locomotor coordination. Furthermore, the types of activities proposed, games, sports, and dynamics involving teamwork and object manipulation, and, to a lesser extent, individual modalities, respond to this approach. This is consistent with the study by Opstoel et al. [35], which found that ball sports improve object control and locomotion, while individual sports benefit locomotion primarily.

Regarding sex, boys perform better than girls, as has been shown in other studies [20,34,36]. However, there is still no consensus, as there are studies in which boys perform better in visuo-motor coordination and worse in locomotor coordination than girls [8].

In relation to sports practice, the data show that children who practice sports obtain significantly higher scores in visuomotor coordination compared to those who do not practice sports [37], agreeing with the results of the study by Rosa Guillamón et al. [34] which indicate that students who perform physical activity have higher values in object control, control of objects with the foot and control of objects with the hand. This lack of consensus may be due to the fact that this study did not take into account the same sports practice profile in the analyzed sample [28].

A significant interaction between sex and age is also observed in visuomotor coordination, showing that boys outperform girls at all ages analyzed. Furthermore, in the interaction between sex and sports practice, boys present higher values than girls, which could be explained by the physical activity and sports preferences of both sexes [38], and where boys show a preference for competitive sports and the expectations of being professional athletes [39], while girls show a greater predilection for modalities related to body expression [40], which opens new lines of research to explore the underlying mechanisms and compare these sports habits with those reported in other contexts and population groups.

On the other hand, in the interaction between age and sport, regarding visuomotor coordination, 6-year-old students, whether they practice sports or not, present lower scores than the rest of the ages, as in the study by Cenizo-Benjumea et al. [8], establishing, again, this age as a reference in the evolutionary calendar of the development of visuomotor coordination [26], as occurred in locomotor coordination, being able to affirm that in addition to the influence of musculoskeletal and nervous system maturation [2], motor experiences are necessary to produce improvements in visuomotor coordination [27], as well as the profile of the sports practice performed [28].

In this study, significant differences were observed at all ages between students who practiced sports and those who did not, with those who did obtaining higher scores, except in the 10-year-old group. This could indicate the existence of a sensitive phase in Primary Education, since the scientific literature itself identifies two key stages in motor development: a general stage, spanning from 7 to 10 years of age, and a specific stage, from 10 years of age (11 to 13), in which a substantial improvement in segmental functionality occurs in object manipulation [41,42]. These findings also suggest that the type of sport practice or modality should be taken into account.

Significant differences were found in the interaction between sex, age, and sports practice, with boys who participate in sports obtaining higher scores at all ages than girls, unlike boys who do not participate in sports. These results are consistent with those described by Müller et al. [36], who found that boys perform better than girls in object control within real motor competence, and that, furthermore, participation in sports activities is associated with higher levels of real motor competence. On the other hand, it is worth highlighting that in the comparison between girls who practice sport and those who do not, significant differences only appear in 6-year-old girls, which suggests that from this age onwards, both the degree of maturation [2] and motor experiences [27] play a relevant role and, at this initial stage, the opportunities for motor development seem to be similar for both sexes [18], differing later depending on the sport practice, age and gender.

#### 4.2.1. Foot Object Control Coordination

Regarding age, a significant increase in FOCC scores is observed as students grow. Regarding gender, boys obtain higher values than girls [20,25,34]. In the study by Andrade-Lara et al. [43], the results also show an upward trend with age, and present greater accuracy and fewer errors in tasks related to eye-foot coordination, so it is possible that this fact is due to preferences for physical activity and sports among boys and girls [38].

Regarding sports practice, the results show that children who participate in sports obtain significantly higher scores on the FOCC than those who do not participate in sports. In this regard, Rosa Guillamón et al. [34] establish a positive association between physical activity and sports practice. This improvement may also be due to the type of sport practiced, which is predominantly a team sport and involves the use of mobile devices [28,35].

Finally, a statistically significant interaction between sex and sport practice was detected, with boys who practice sports scoring higher than girls on the FOCC. This could be because girls tend to drop out of sport more frequently with increasing age, compared to boys who show greater interest in sport [44,45].

Regarding the FOCC and its interaction between sex, age, and sports practice, the results show that boys who practice sports obtain higher scores than girls, regardless of the age group analyzed. This finding suggests that, although sports practice benefits both sexes, boys tend to achieve higher performance in this capacity throughout the different stages of Primary Education, which may be influenced by biological differences [17], sports preference [46], the type of sports modality [36], levels of practice [47], or the degree of sports specialization [48].

#### 4.2.2. Hand Object Control Coordination

Regarding Hand Object Control Coordination (HOCC), boys obtain higher scores than girls [8,20,34]. Furthermore, it has been observed that boys obtain higher scores than girls at all ages studied, except at 11 years, where the opposite occurs. These variations may be due to the difference in biological age and maturation between the sexes, since girls show a faster maturation rate than boys [17].

Finally, it has been observed that students who participate in sports perform better than those who do not. In this regard, Rosa Guillamón et al. [34] establish a positive association between physical activity and sports participation.

Regarding the HOCC and its interaction between age and sports practice, students aged 6 and 7, regardless of whether they practice sports or not, present lower scores than other age groups. This finding suggests that the development of HOCC at these early ages may be influenced by the degree of maturation [2] or by limited motor experience [27]. This fact, as with the FOCC, could be related to the existence of a series of sensitive phases within Primary Education influenced by age, since, around 7 years of age, basic motor skills begin to develop, including visuomotor skills and, from 10 years of age (11 to 13 years), specific motor skills specific to the sport [41,42]. In this sense, in the present study, significant differences were observed in the HOCC between students who practice sports and those who do not, in practically all the ages analyzed except in the 9- and 10-year-old groups, where the differences did not reach statistical significance, coinciding with the stage in which specific motor skills begin to be worked on [41,42]. These results reinforce the evidence that sports practice contributes positively to the development of basic motor skills, such as controlling objects with the hand, especially at early ages. However, the absence of significant differences in the 9- and 10-year-old groups could be related to factors such as the variability of the stages of development throughout the specific stage [41,42].

### 4.3. Global Motor Coordination

Regarding the Global Motor Coordination (GMC), the present study observed that it increases significantly with age, a finding that partially coincides with the study by Rosa Guillamón et al. [34]. Although this study only evaluated students between 6 and 8 years old, its results coincide in identifying that older students present the best GMC indices. Taking into account sex, the present study observes that boys’ GMC shows higher values compared to girls [34]. This result contradicts studies that indicate that the evolution of general coordination occurs similarly between boys and girls. However, between 8 and 9 years of age, girls present greater global motor coordination than boys [18], and from 10 years of age onwards, differences in performance begin to appear in favor of boys [8]. Therefore, scientific evidence would need to delve deeper into which maturational capacities influence each phase of development during the school stage, depending on sex [17]. Regarding sports practice, data show that boys and girls who practice sports obtain significantly higher scores in GMC than those who do not participate in sports, which highlights the positive association between physical activity carried out in different sports disciplines and the development of global motor coordination [34].

Older children who participate in sports have been observed to have the highest scores on the GMC, which could be due to the fact that boys participate more in sports activities than girls [17]. However, the level of motor practice generally influences motor coordination [2]. Thus, in other studies where levels of sports practice are not considered, boys do not obtain these superior results in all aspects of coordination [8,19]. In this sense, it is important to ensure that the time devoted to sports practice is not excessive and that it considers the prevention of possible postural alterations that could lead to injuries or pathologies derived from overspecialization [21]. In turn, the 6 and 7-year-old groups that participate in sports, and the 6-year-old group that does not, present lower scores than the rest of the age groups. These findings suggest that, at 6 years of age, both the degree of maturation [2] and motor experiences [27] manifest themselves in a similar way, differing later on [18]. In this sense, in the present study, significant differences were found at all ages (except at 10 years of age) between students who practice sports and those who do not, with higher scores in those who do, indicating that from this age onwards, sports practice begins to play a relevant role [5,49,50].

This study has limitations, such as its cross-sectional design, which prevents establishing causal relationships and analyzing changes over time. Although the study controlled for BMI as a confounder, there were other variables that were not considered to influence the results: (1) The type of sport practiced by the participants, since, for example, team sports (soccer, basketball) may favor more visual-motor coordination and object control (FOCC/HOCC), while individual sports (gymnastics, athletics) could influence more locomotor coordination (LC); (2) Neither the frequency nor the intensity of sports practice (number of weekly hours dedicated to sport, which could modulate the magnitude of the differences observed between practitioners and non-practitioners) were recorded. A greater training load could be associated with better motor development [48]. (3) Biological maturation can also be considered a confounding variable since, although chronological age was controlled, pubertal stage was not evaluated, which varies between individuals of the same age and affects motor development [12,17]. (4) Finally, the variables of the quality of physical education classes, the number of weekly sessions, or the pedagogical approach were not analyzed, which could affect motor development depending on the educational center [5,34]. Furthermore, the sample is not representative of the entire Spanish school population, which restricts the generalizability of the findings. Sociocultural or motivational factors that could explain the differences between genders and ages were not examined in depth. Finally, although standardized tests were used, factors such as prior learning, the evaluator, or specific motivation may influence the reliability of the scores. It is recommended that the results be interpreted with caution and that future longitudinal studies be conducted with more representative samples.

Future research should explore comparing the sports disciplines practiced by participants to identify optimal activities for skill development. For example, comparing the effects of team sports (e.g., soccer) with those of individual sports (e.g., track and field) on the skills studied; quantification of the frequency, duration, and intensity of practice should also be studied. Quantification of these variables (e.g., through accelerometry or training logs) would clarify dose-response relationships.

Furthermore, future use of longitudinal designs would allow for annual monitoring of motor development to establish causal pathways, such as incorporating school-level factors (e.g., quality of the physical education curriculum, teacher training, etc.) or including biological markers, where hormonal or skeletal maturity is taken into account to control for maturation effects, to separate age from maturation effects.

This study expands our understanding of the determinants of motor coordination, but underscores the need for more nuanced and contextualized research. By addressing these limitations, future work can optimize interventions to ensure equitable motor skill development for all children. Clinicians and educators should interpret these findings as preliminary evidence to guide, not prescribe, practice, pending further validation.

## 5. Conclusions

Below, we present the conclusions of this study, integrating methodological limitations and future lines of research to improve its clinical and educational applicability:

1. Motor coordination skills (LC, VC, FOCC, HOCC, and GMC) improve significantly with age, especially in visuomotor coordination (VC) and hand-object control coordination (HOCC). This is consistent with neurodevelopmental theories that suggest a progressive refinement of motor skills during Primary Education. Therefore, as practical implications, it should be noted that schools should implement age-specific motor skill development programs, with an emphasis on VC and HOCC in the older grades (9–11 years). Early intervention (6–8 years) can address deficiencies in fundamental skills such as locomotor coordination (LC).
2. Boys outperformed girls in VC, FOCC, HOCC, and GMC, whereas no gender differences were observed in LC. This suggests that gender differences are task-specific, possibly influenced by activity preferences (e.g., boys may participate more in ball sports) and sociocultural factors (e.g., gender-differentiated play opportunities). Based on these results, it is suggested that physical education curricula should promote inclusive activities to reduce gaps in object control skills. Furthermore, girls’ participation in FOCC/HOCC-oriented sports (e.g., soccer, basketball) should be encouraged.
3. Children who participated in sports showed superior motor coordination across all variables studied, with notable benefits in CV and FOCC. Structured physical activity appears to be an important factor in the development of complex skills such as visuomotor integration. Policy implications include the integration of extracurricular sports into school curricula, especially for disadvantaged populations. In practice, sports with high visuomotor demands (e.g., tennis, basketball) should be prioritized in physical education.

## Figures and Tables

**Table 1:** 3JS Test [7,22].

Test Dimension (Range)	Sub Test (Range)
Locomotor coordination (LC; 3–12)	1st Vertical jump (1–4)
	2nd Rotation about the longitudinal axis (1–4)
	3rd Slalom race (1–4)
Visuomotor coordination (VC; 4–16)	Foot object control coordination (FOCC; 2–8) 4th Precision strike (1–4) 5th Dribbling the ball + slalom (1–4)
	Hand object control coordination (HOCC; 2–8) 6th Precision throw (1–4) 7th Bounce the ball + slalom (1–4)
Global motor coordination (GMC; 7–28)	LC + VC

**Table 2:** Sample characterization regarding height, weight, and BMI.

		Height (m)	Weight (kg)	BMI
	Age	M ± SD	M ± SD	M ± SD
Boys	6	1.19 ± 0.05	22.48 ± 4.09	15.69 ± 2.24
	7	1.25 ± 0.05	27.01 ± 4.99	17.06 ± 2.41
	8	1.31 ± 0.07	31.26 ± 8.86	17.91 ± 3.53
	9	1.35 ± 0.06	32.68 ± 6.10	17.64 ± 2.56
	10	1.44 ± 0.07	39.83 ± 8.27	19.05 ± 2.87
	11	1.49 ± 0.07	43.53 ± 7.95	19.35 ± 2.84
Girls	6	1.20 ± 0.05	23.49 ± 4.04	16.06 ± 2.00
	7	1.23 ± 0.05	28.08 ± 6.29	18.23 ± 3.32
	8	1.30 ± 0.07	29.85 ± 6.77	17.47 ± 2.74
	9	1.35 ± 0.07	32.82 ± 6.38	17.78 ± 2.75
	10	1.41 ± 0.07	39.34 ± 8.69	19.51 ± 3.37
	11	1.48 ± 0.08	42.05 ± 7.15	19.02 ± 2.71

The reliability analysis yielded adequate results for the variables studied (McDonald’s Omega > 0.700), locomotor coordination (Omega = 0.701), visuomotor coordination (Omega = 0.824), and global motor coordination (Omega = 0.849).

**Table 3:** Locomotor coordination results of the 3JS test based on age, gender, and sports practice.

		Boys		Girls		Main Effects			Interactions
	SP	Yes	No	Yes	No	Gender F	Age F	SP F	F
	Age	M ± SD	M ± SD	M ± SD	M ± SD				
LC (3–12)	6	8.00 ± 2.23	6.92 ± 1.55	8.29 ± 2.16	6.21 ± 2.50		29.908 ***	18.586 ***	(1) 2.420 *
	7	9.57 ± 1.79	8.40 ± 0.54	8.97 ± 2.17	8.71 ± 1.93				
	8	10.08 ± 1.59	9.42 ± 1.78	9.10 ± 1.57	9.52 ± 1.94				
	9	10.52 ± 1.57	9.71 ± 1.67	9.54 ± 1.83	9.72 ± 1.56				
	10	10.13 ± 1.25	9.55 ± 1.81	9,73 ± 1.64	9.25 ± 1.86				
	11	10.52 ± 1.59	8.40 ± 2.40	9.81 ± 1.65	8.77 ± 1.48				

Note. M = Mean; SD = Standard deviation. SP = Sport practice; LC = Locomotor coordination. * *p* < 0.05; *** *p* < 0.001; Interactions (1) Age × Sports Practice.

**Table 4:** Visuomotor coordination results of the 3JS test based on age, gender, and sports practice.

		Boys		Girls		Main Effects			Interactions
	SP	Yes	No	Yes	No	Gender F	Age F	SP F	F
	Age	M ± SD	M ± SD	M ± SD	M ± SD				
VC (4–16)	6	9.40 ± 2.35	6.30 ± 2.39	7.59 ± 2.22	5.21 ± 1.22	73.931 ***	47.393 ***	72.313 ***	(1) 11.744 * (2) 19.652 *** (3) 2.290 * (4) 2.560 *
	7	11.09 ± 2.39	7.20 ± 0.83	8.15 ± 2.02	7.87 ± 1.92				
	8	12.42 ± 2.32	10.78 ± 2.22	9.60 ± 2.24	9.05 ± 2.43				
	9	13.36 ± 2.45	11.52 ± 2.31	10.40 ± 2.24	8.88 ± 2.49				
	10	13.54 ± 1.77	12.00 ± 2.54	10.40 ± 2.01	9.95 ± 2.30				
	11	13.74 ± 2.38	8.60 ± 2.19	10.46 ± 2.13	10.22 ± 2.48				

Note. M = Mean; SD = Standard deviation. SP = Sport practice; VC = Visuomotor coordination. * *p* < 0.05; *** *p* < 0.001; Interactions: (1): Gender × Age; (2) Gender × Sports Practice; (3) Age × Sports Practice; (4) Gender × Age × Sport Practice.

**Table 5:** Foot object control coordination results of the 3JS test based on age, gender, and sports practice.

		Boys		Girls		Main Effects			Interactions
	SP	Yes	No	Yes	No	Gender F	Age F	SP F	F
	Age	M ± SD	M ± SD	M ± SD	M ± SD				
FOCC (2–8)	6	5.08 ± 1.54	3.38 ± 1.60	3.62 ± 1.11	2.52 ± 0.61	26.069 ***	-	59.431 ***	(2) 18.528 *** (4) 2.2742 *
	7	5.59 ± 1.34	3.60 ± 0.89	3.65 ± 0.90	3.71 ± 1.13				
	8	6.37 ± 1.23	5.42 ± 1.34	4.71 ± 1.31	4.23 ± 1.60				
	9	6.65 ± 1.41	5.85 ± 1.15	4.92 ± 1.21	4.00 ± 1.18				
	10	6.81 ± 1.05	5.66 ± 1.65	4.82 ± 1.26	4.60 ± 1.53				
	11	6.92 ± 1.39	4.20 ± 1.30	4.62 ± 1.48	4.66 ± 1.22				

Note. M = Mean; SD = Standard deviation. SP = Sport practice; FOCC = Foot object control coordination. * *p* < 0.05; *** *p* < 0.001; Interactions: (1): Gender × Age; (2) Gender × Sports Practice; (3) Age × Sports Practice; (4) Gender × Age × Sport Practice.

## Data Availability

The data presented in this study are not available in accordance with Regulation (EU) of the European Parliament and of the Council 2016/679 of 27 April 2016 regarding the protection of natural persons with regard to the processing of personal data and the free circulation of these data (RGPD).

## Abbreviations

The following abbreviations are used in this manuscript:

MC	Motor coordination
LC	Locomotor coordination
VC	Visuomotor coordination
FOCC	Foot object control coordination
HOCC	Hand object control coordination
GMC	Global motor coordination
BMI	Body Mass Index
MANCOVA	Multivariate analysis of covariance
